# Design and maintenance of medical oxygen concentrators in Sub-Saharan Africa: a systematic review

**DOI:** 10.1186/s12913-025-12315-6

**Published:** 2025-01-29

**Authors:** Nahimiya Husen Ibrahim, James Wallace, Davide Piaggio, Leandro Pecchia

**Affiliations:** 1 Department of Engineering, University Campus Bio-Medico of Rome, Via Alvaro del Portillo 21, Rome, 00128 Italy; 2 School of Engineering, University of Warwick, Library Rd, Coventry, CV4 7AL UK

**Keywords:** Oxygen concentrator, Low-resource settings (LRSs), Maintenance, Oxygen supply, Frugal design

## Abstract

**Background:**

Oxygen therapy is critical and vital treatment for hypoxemia and respiratory distress, however, access to reliable oxygen systems remains limited in SSA. Despite WHO initiatives that distributed over 30,000 OC oxygen concentrators worldwide, SSA faces significant challenges related to their maintenance and use, due to harsh environmental conditions, technical skill shortages and inadequate infrastructure. This review aims to systematically identify and assess the literature on OC design adaptations, maintenance challenges, and knowledge gaps in SSA, providing actionable recommendations to inform innovative and context-sensitive solutions to improve healthcare delivery in the region.

**Methods:**

The study focused on medical oxygen concentrators in SSA countries. It was conducted by following the PRISMA statement and searching three databases, i.e., Scopus, PubMed, and Web of Science, for publications in the period 2001–2023, using the search terms: oxygen concentrator, therapy, cylinder, plant, supply, delivery, and availability, design, and maintenance. The screening process involved evaluating manuscripts based on their titles, abstracts and full texts, based on specific inclusion and exclusion criteria. The extracted information included the author’s publication year, country, study aim, and key findings.

**Results:**

Overall, 1,057 papers were returned for our analysis, of which 20 met the inclusion criteria. These studies primarily examined the design, availability and cost-effectiveness of oxygen concentrators compared to cylinders, revealing a significant supply and demand gap for these devices in SSA. It also illustrated how the environmental challenges impacted the devices durability, highlighting the need for more locally adapted resilient solutions. Solar-powered systems provide a sustainable option in areas with unstable power supplies, although initial costs remain high. Robust maintenance strategies, capacity building and strict procurement protocols proved essential to ensuring equipment long-term functionality.

**Conclusion:**

This review synthesized and critically assessed the current in the body of literature, enabling highlighting valuable insights for innovators and stakeholders with an interest in enhancing the oxygen availability in SSA. It highlighted a pressing need for improved healthcare infrastructure investment, context-aware OC design and novel standards and regulatory frameworks to support frugal innovation.

**Supplementary Information:**

The online version contains supplementary material available at 10.1186/s12913-025-12315-6.

## Background

Oxygen is an essential medicine – lifesaving for many pathologies involving the respiratory distress of patients [1]. Since the late 1800s, oxygen has been utilized as a crucial and lifesaving medical therapy for treating both acute and chronic conditions that result in hypoxemia, an abnormally low level of oxygen in the blood. Oxygen was listed as one of the World Health Organization (WHO) Essential Medicines for the management of hypoxemia in 2021 [2].

With the aim of improving access to oxygen, as of February 2021, the WHO and its collaborators had succeeded in disseminating more than 30,000 OCs among 121 countries. Thirty-seven of these countries were classified as “fragile” and the support provided included technical advice and, in some cases, the procurement of oxygen at scale [3]. In fact, the WHO and partners have been collaborating to support the establishment of functional oxygen system infrastructures in Low-Resource Settings (LRS) including Sub–Saharan African (SSA) countries’ health care facilities.

This is not an easy task, as in general, medical devices (MD) in SSA countries, as well as in other LRSs, tend to malfunction or break down quite frequently: a comprehensive analysis of over 100,000 pieces of equipment found that the percentage of nonfunctioning MD in low- and middle-income countries (LMICs) ranged from 0.83% to 47% [4–6]. The causes of this are related to the challenges that affect these settings, including the lack of medical and technical expertise, the non-functional health technology management, a poor supply chain, and the harsh local working conditions (e.g., excessive temperatures and humidity, unstable power supply, etc.) [4, 7, 8]. As a result, local biomedical engineers and technicians typically have to reallocate the already scarce resources to repair or dispose of donated equipment [9–12]. This hinders the equitable access to healthcare in favour of Higher-Resource Settings (HRSs) and High-Income Countries (HICs), with those necessitating healthcare access the most, i.e., a wide majority of the world’s population typically living in LRSs, being the ones who benefit less from it [11].

In the numerous and variate family of MDs, electromedical devices are those suffering the most from the aforementioned challenges, including OC. An OC is a compact electrical device used to extract oxygen from atmospheric air, by filtering out nitrogen [13, 14]. It is the preferred source of oxygen for hospitals and home use in long-term continuous oxygen therapy, especially in remote and low-resource areas lacking oxygen facilities and cylinder supply networks. Gas cylinders have previously been the main source of oxygen in LMICs; however, they are costly to refill and challenging to transport, particularly to remote health care facilities, making them unsuitable within these settings [15–17].

A study set in Kenya, exploring oxygen availability within healthcare facilities, reported that out of 57 patients assessed, 18 (32%) faced oxygen interruptions due to power outages with a median duration of 11 min. This contributed to the case fatality rate of 19% amongst the affected patients [18]. Another study by Belle et al. reporting on influenza preparedness across 230 facilities in LRSs claimed that 99 (43.8%) had uninterrupted access to an oxygen source, 71 (31.4%) had oxygen sometimes available and 56 (24.8%) did not have any oxygen source available. It also found that 75.5% did not have access to or only sometimes had access to OC [19], highlighting The importance of reliable infrastructure for the delivery of effective healthcare within LRS. Despite the key role that OCs play in LRSs and the related challenges, there is limited data about their specific maintenance practices and design features, as well as their specific challenges, especially for those used in LRSs.

In fact, several studies assessed the availability of equipment in healthcare settings across SSA [12, 20–23]. Some of these focused on MDs in LRSs, and most of them focus on oxygen therapy for lower respiratory disease and oxygen availability in healthcare [24–27].

One of the main issues reported by Piaggio et al. in several publications relates to the inadequacy of the current standards and regulations behind MD, which are often too strict and do not take into consideration each particular context of LRSs [28–30]. Therefore, when MD designed for up-to-standard locations end up being used in a LRSs of a LMIC, where such standard conditions are far from being ideal, they easily break down or malfunction, or they may not even be safe to use. This is especially true for delicate pieces of medical equipment, such as OCs. To this regard, in another publication, Piaggio et al. described how the harsh environmental conditions summed up with other local challenges had hindered the safe and effective working of an OC in Benin, whose filters had never been changed and had been working over the limit suggested by the manufacturer when used in standard conditions. One exemplary study by Williams et al., following a frugal engineering approach, attempted to solve a common issue of OCs, that of the exhaustion of the inlet filters and the poor supply chain. In particular, they applied reverse engineering principles and redesigned the filter so that it could be 3D printed and locally manufactured relying on activated charcoal as a filtering agent, reaching mediocre results [31].

Therefore, more research should be carried out on designing devices to withstand these challenges, focusing on designing more resilient devices through the application of new innovative materials and more adaptive technologies that would be more suitable. It is most important, though, that local contexts and local communities are taken into consideration and involved to make sure that the resulting device is contextualised and aligned with the local culture, promoting at the same time capacity building and knowledge exchange [29, 32, 33].

In this remit, given the scarcity of evidence related to OCs in SSA and LRSs, it is paramount to collect and assess the available evidence in order to have a better understanding and inform the design of tangible solutions, with a specific focus on the cost-effectiveness of various oxygen delivery systems within LRSs, and the development of a better understanding of the long-term maintenance requirements to improve the life span of existing concentrators.

The goal of this systematic review is, therefore, to assess what is currently known in the literature about possible resilient design modifications for OCs, their maintenance challenges, and knowledge gaps, for their use in SSA as well as to make recommendations for future researchers.

This work aligns with two latest WHO publications, i.e., the “Compendium of innovative health technologies for low-resource settings” and “Medical device donations: considerations for solicitation and provision”, on which we had the pleasure of collaborating [34, 35].

## Methodology

This study used a systematic literature review approach to identify, critically appraise, and synthesize existing research on the challenges in the design and maintenance of OCs in SSA. The review was carried out according to the PRISMA guidelines [36].

### Search strategy

Three electronic databases Scopus, PubMed, and Web of Science were selected for the search. A comprehensive search string was developed using Boolean operators to combine the following terms: “OC,” “Oxygen Therapy Device,” “Oxygen Cylinder,” “Oxygen Plant,” “Concentrator Oxygen Supply,” “Medical Oxygen,” “Clinical Oxygen,” “Oxygen Delivery,” and “Oxygen Availability,” along with the names of all SSA countries. The string was developed in a series of focus groups with biomedical and clinical engineers. The full search string is provided in the Supplementary materials (see Additional file 1).

### Selection procedure

Search results were exported to an Excel sheet, including titles, abstracts, publication years, and other relevant data. Three authors were independently involved in the screening process, which followed a by title, by abstract and by full text approach. The third reviewer resolved possible discrepancies in the selection choice between the other two, to ensure consistency and bias mitigation.

### Eligibility criteria

Studies were included if they focused on medical oxygen systems (e.g., concentrators, cylinders, plants) in LRS, specifically SSA, were published in English, and were original research articles. Studies conducted outside LRSs, non-peer-reviewed articles, and those focusing on unrelated MD (e.g., anaesthesia machines, mechanical ventilators) were excluded. The review included studies published from 2001 onwards, as this period marked advancements in OC technology and increased global attention to oxygen availability in LRSs.

### Quality assessment

To ensure the reliability and validity of the included studies, a quality assessment was performed based on the Mixed Methods Appraisal Tool (MMAT) [37]. This tool is widely used tool designed for the quality assessment of empirical studies in systematic reviews, particularly when the review includes studies with diverse methodological approaches. Both qualitative and quantitative aspects were evaluated, including the appropriateness of the research approach, adequacy of data collection methods, sampling strategy, representation of the target population, and the appropriateness of the statistical analysis.

### Data extraction and synthesis

Relevant data, including author names, publication years, study objectives, and key results, were extracted into a standardized Excel spreadsheet. The narrative synthesis approach [38] was then systematically applied to identify and integrate themes, patterns and relationships across the studies. This process involved several steps: first, a thorough review of the extracted data was conducted to ensure familiarity and context. A methodical categorization process used Excel to find recurring themes and draw important conclusions from the data. These ideas were then grouped into general themes by contrasting their similarities and differences across the extracted information. These codes were then grouped into overarching themes by comparing similarities and differences across the data. Relationships between themes were examined using a thematic matrix that enabled mapping of relationships and identification of contextual influences. Finally, the themes and their relationships were synthesized into a coherent narrative that made it possible to draw meaningful conclusions and provide a solid foundation for the research findings.

## Results

### Search results

**Fig. 1:**
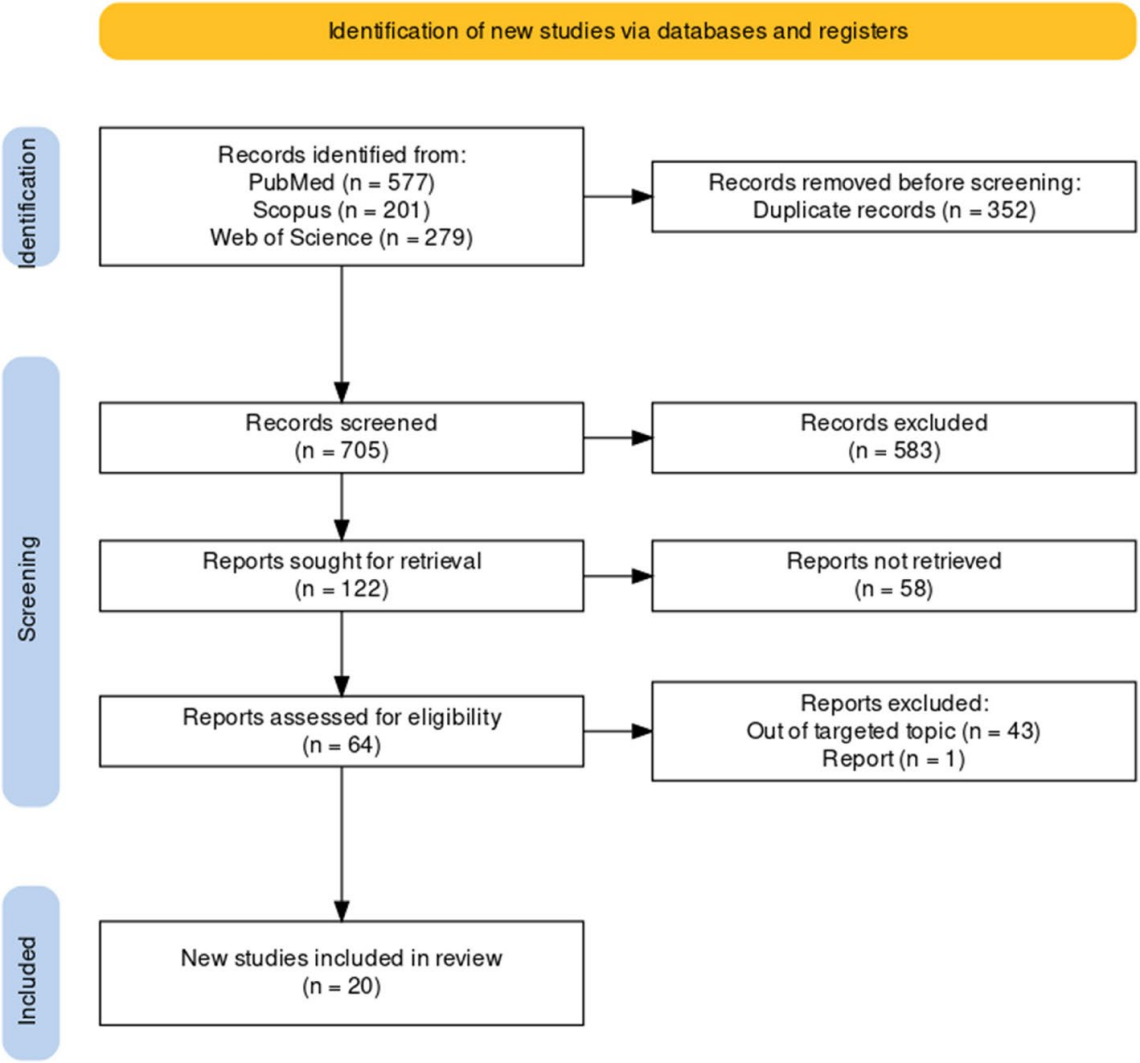
PRISMA flow chart

In Fig. 1 the search result is presented. Initially, the combined search on the three databases returned 1057 hits. After duplicates were removed, there was a total of 705 records to screen. The screening and quality assessment process ended with a total of 20 studies to be included in this review. All included study met “YES” (see Additional file 2) criteria for the relevance of MMAT checklist domain, ensure that no articles were excluded due to poor quality.

### Data extraction

The included studies were categorized into three major themes, namely Maintenance, Frugal design, and Oxygen availability and cost effectiveness. The following sections report details according to one of the above themes.

### Maintenance

Four studies (see Table 1) from Gambia, Nigeria, Uganda, Kenya, and Malawi were analysed to explore maintenance challenges in SSA. These countries were selected because they represent diverse geographic, economic, and healthcare system contexts within SSA and are the ones who documented challenges and innovations in OC maintenance and design via scientific publications.

**Table 1:** Reports the extracted information about the studies focused on maintenance practices

Author/s	Objectives	Result/Main finding
Bradley et al., 2015 Gambia [15]	To assess the long-term reliability and maintenance needs of OC in low-income settings with biomedical engineering technologist support	27 OC 5-year maintenance data included in the analysis. The mean age of the OCs was 6.1 years, and the mean total hours of use was 6,267, with annual use per device being approximately 1,480 h. Most concentrator faults were resolved within a day, and resolving multiple component failures such as compressor failures, molecular sieve bed saturation and printed circuit board (PCB) requires more time and budget. Most failures were repaired for less than $10 and required little experience
Graham et al., 2020 Nigeria/Uganda/Kenya [39]	Field experience to help Hospitals to improve their Oxygen supply system by using Low-cost technology and sharing guidance documents for local use and adaptation	Assist biomedical engineers and technicians in improving existing oxygen supplies through training, protocol and logistical support. Expanding existing oxygen systems through robust equipment and intelligent design, cost-effective OC-based systems relying on simple plastic tubing and flowmeter stands to deliver oxygen to multiple patients simultaneously
Vincente et al., 2011 Malawi and (Mongolia) [40]	To describe OC functioning in two countries with widespread, long-term use of concentrators as a primary source of oxygen for treating children	28/36 OCs that were installed in Malawi for 48 months were functional, and 13/25 of the OCs installed in Mongolia for 36 months were functional. For the first 30,000 h, all OCs installed were functional and performance varied from brand to brand. However, resources for maintenance are limited and therefore there are several OCs that are not functional
Enarson et al., Malawi 2008 [16]	To validate five key steps to introduce and implement OCs in Malawi	Feasibility was assessed to implement an oxygen system using concentrators throughout a low-income country. Oxygen delivery requires trained staff with necessary equipment and supplies. Regular maintenance and supervision are essential to ensure optimal utilization

Key findings from the analysis of maintenance challenges in SSA highlight significant issues and potential solutions for the sustainability of OCs. Studies from Gambia, Nigeria, Uganda, Kenya and Malawi show that while simple maintenance tasks, such as minor repairs, can often be completed within a day at minimal cost (less than $10), complex failures in components such as compressors, molecular sieves beds and PCBs require longer repair times and higher budgets [15, 39, 40].

For instance, Bradley et al. (2015) found that 27 OCs in The Gambia had a median age of 6.1 years and a median total service life of 6,267 h, with most malfunctions resolved quickly. However, multiple component failures resulted in increased downtime and higher costs, highlighting the need for preventive maintenance and robust repair protocols. Similarly, Vicente et al. (2011) reported that while a significant proportion of OCs remained functional after several years of use, performance varied across brands and limited maintenance resources often resulted in non-functional units. To address these challenges, Graham et al. (2020) and Enarson et al. proposed several innovative solutions, including the use of flow dividers to allow oxygen delivery to multiple patients simultaneously, surge protectors to protect equipment, and training programs for biomedical engineers.

Additionally, the integration of central piping systems with flow meters in Nigerian hospitals and the establishment of intensive care units in Malawi for intensive care patients are examples of successful design changes that have resulted in improved efficiency and reliability. Despite these efforts, critical gaps remain, including the lack of long-term performance data, limited access to replacement parts, and the need for cost-effective, local maintenance solutions. Additionally, studies show significant variation in the quality of OCs used in low-resource environments, highlighting the importance of standardizing equipment to ensure reliability and simplify maintenance.

These findings emphasize the necessity of long-lasting, straightforward designs and environmentally friendly maintenance practices. Instances like the successful implementation of cost-effective preventive maintenance procedures in The Gambia and the efficient use of pulse oximetry to optimize oxygen consumption in Uganda and Nigeria demonstrate encouraging directions for the future. In environments with limited resources, OC systems can be made more resilient by filling in gaps and using holistic approaches.

### Frugal design concept for oxygen concentrator

In order to increase the robustness and resilience of the OCs when deployed in difficult environments, five studies (see Table 2) concentrated on cost-effective solutions and design modifications.

**Table 2:** Study focuses on the system design of OC

Author/Year/Country	Objectives	Result
E. Williams et al., 2021 LRSs [31]	To re-design a frugal solution for OCs inlet filters	The prototype, which is based on 3D printing and activated charcoal, can be locally produced and has filtering efficiency of 64.2% for particle greater than 1um, and of 38.8% for particles greater than 0.3um
Rassool et al., 2017 LRSs [14]	To design and test a prototype of a low-pressure oxygen storage system	Flow of 1.2 L/min was continuously kept to a simulated patient during 30 days on grid power, despite power failures totalling 2.9% of the total time, with durations of 1–176 min (mean 36.2, median 18.5)
Turnbull et al., 2016 Uganda [43]	To design and pilot a solar-powered oxygen delivery system	The resulting system has an output of up to 5 l/min flows of oxygen at 88% [± 2%] purity A group of 28 patients with hypoxaemia were successfully treated from October to December with 13 power outages, corresponding to 10% of test time without electric power
Calderon et al.,2019 Uganda [42]	To evaluate a prototype device performance measure in the lab and in a hospital setting	The 75-day trial was conducted at Jinja Regional Referral Hospital. The unit flow rate was adjusted between 0.5 and 5 throws per minute once per week. During the test period, 1,284 power outages were recorded with an average duration of 3.1 min (range: 0.08 to 1,720 min). Over the course of the study, the low-pressure system was able to provide oxygen for 56% of the 4,295 min of power outages and covered over 99% of power outage events
Otiangala et al., 2021 Kenya [56]	To design and test a medium pressure reservoir (MPR), also studying its usability in a controlled pre-clinical study	This study reported the deployment of medium pressure regulators (MPR) to limit oxygen scarcity due to power outages. They found that within 2 facilities power was unavailable for a median of 4.6 min with an IQR of 3.6 to 11 min accounting for 2% of the total time the facility’s were observed. 881, 445, and 178 people could receive a continuous flow rate of 1, 2, 5 LPM from the MPR at a storage pressure of up to 790 kPa. Positive comments were received from users, who said it is simple to use and helpful in an emergency

Williams et al., in their paper, designed, prototyped and an inlet filter for OCs, relying on reverse engineering principles and 3D printing, using locally available and producible materials, specifically activated charcoal, as a filtering agent [31]. They tested this newly designed filter in a laboratory setting, and the results were promising: the filtering efficiency for particles greater than or equal to 1 micron, and for those greater than or equal to 0.3 microns in size, were measured at 64.5%, and 38.8%, respectively. These numbers were lower when compared to the original filter performance, set at 96.3% for particles greater than or equal to 0.3 microns. Nonetheless, the authors argue that this is a satisfactory result, because the aim of the inlet filter is filtering out the gross particles to provide a cleaner airflow to the OC. Moreover, using this novel filter, which is easy to produce and service in loco, would indefinitely extend the lifespan of the output filter, as well as of the OC, when compared to its use without any inlet filter at all, or with an overused paper-based one (original).

These two studies [41, 42] collectively emphasize the importance and feasibility of low-pressure oxygen storage systems for maintaining continuous oxygen supply in environments with unreliable power supplies and highlight their potential to reduce infant mortality due to pneumonia in LRSs. The authors have demonstrated the feasibility and functionality of an oxygen storage system designed for emergency situations, particularly in rural areas where the electricity supply is not continuously guaranteed. In a study conducted in Mbarara, a low-pressure oxygen storage system was designed and tested to maintain a continuous flow of 1.2 Liters per minute to a simulated patient over a period of 30 days using mains power. Despite power outages, which accounted for 2.9% of the total time and lasted between 1 and 176 min (with a mean of 36.2 and a median of 18.5), the system proved to be effective. Feasibility assessments were also carried out at Jinja Regional Referral Hospital and produced promising results. In a trial lasting 2 months and 15 days, the flow rate was adjusted weekly between 0.5 and 5 Liters per minute and 1,284 power outages with an average duration of 3.1 min (ranging from 0.08 to 1720 min) were recorded. Throughout the study, the low-pressure system successfully delivered oxygen during 56% of the 4,295 min power outages and covered over 99% of the power outages. Such systems can significantly improve the treatment of childhood pneumonia and other diseases requiring oxygen therapy and ultimately reduce the mortality rate in LRS.

Turnbull et al. developed and tested a solar-powered oxygen delivery system for paediatrics pneumonia in a Ugandan hospital. The system used 25 solar panels, a battery bank and a 300W OC. In a pilot study of 28 children, the system improved oxygen saturation by an average of 12% and reduced disease severity. Although power reliability was an issue during extended cloudy periods, the results were promising and suggest that solar energy is a viable solution for oxygen therapy in resource-limited environments [43, 44]. However, the initial cost of purchasing and installing the system in a resource-limited area is still a challenge unless stakeholders and donors expand their support.

### Oxygen device availability and cost effectiveness

Eleven studies relate to the availability of OCs and their cost-effectiveness (see Table 3).

**Table 3:** Summary of oxygen availability and cost effectiveness

Author	Objectives	Result
Stephen RC Howie et al. 2009 Gambia [50]	To compare functionality and cost of oxygen cylinder versus OC	The study found that OCs are beneficial in environments with reliable power supply due to lower operating costs, primarily due to electricity costs, while oxygen cylinders are preferable in locations with unreliable power supply but possible transportation. Only two of 12 Gambian health facilities were suitable for concentrators; Cylinders were better suited for the rest
Otiangala et al. 2020 Kenya [18]	To assess the availability of therapeutic oxygen and evaluate the reliability of the electrical supply and investigate the effects of suboptimal oxygen delivery on patient outcomes	Two of 11 facilities had no oxygen equipment, and nine facilities had at least one concentrator or cylinder. The facilities experienced an average of seven power interruptions per week (range: 2–147). The average duration of the power outage was 17 min and the longest was more than 6 days
Tolla et al. 2022 Ethiopia [46]	Decentralizing oxygen availability and use at primary care level for children under-five with severe pneumonia	Regarding the availability of oxygen equipment (e.g. cylinders, concentrators, and pulse oximeters) in the facilities, seven (58%) facilities did not have any at baseline, but due to the interventions, all facilities were equipped with oxygen equipment
Tolla et al. 2021 Ethiopia [57]	To assess changes in the functional availability of oxygen devices and clinical practices of oxygen therapy in in-patient paediatrics department from public hospitals in Ethiopia during the implementation of interventions	The study was conducted in 32 Ethiopian public hospitals where capacity building and technical support activities were implemented. Of these 32 facilities, 15 (46.9%) were general hospitals, 10 (31.2%) were referral hospitals, and 7 (21.9%) were primary hospitals. Functional oxygen availability has shown a statistically significant increase from 62 to 100% in paediatrics inpatient wards of general and referral hospitals (*p*-value < 0.001)
Nabwire et al., 2018 Uganda [47]	To assess the availability and functionality of oxygen delivery equipment and evaluate nurses’ knowledge and practices with respect to oxygen delivery at paediatric wards of district hospitals in eastern Uganda	Functional oxygen support was available in the paediatric wards of only 2 of 11 (18%) hospitals. Of the 6 concentrators found, two did not work at all and two produced a stream of O2
Huang et al., 2021 Uganda [48]	To assess a solar-powered oxygen delivery (solar-powered O2) a cost-effectiveness and intervention for use in children younger than 5 years with hypoxemia in LRSs	The 10 Year Horizon study compared the costs and health outcomes of solar-powered O2 to (1) zero-case oxygen, (2) grid-powered OCs, and (3) fuel generator-powered concentrators. The use of solar-powered O2 was cost-effective compared to zero-case and grid-powered concentrators and cost-saving compared to fuel generator-powered concentrators
Bradley et al., 2016 Gambia [49]	To identify and isolate the oxygen supply problem in The Gambia by testing a solution at the Medical Research Council MRC Hospital	The new system saved at least 51% in oxygen supply costs compared to cylinders, although the savings due to cylinder leaks were likely far greater. Users reported that the system was more user-friendly and reliable, although technical support and staff training were still required
Mokuolu & Ajayi, 2002 Nigeria [17]	To describe the experiences with an OC in a neonatal unit and economic evaluation methods to demonstrate its cost-effectiveness	The cost of using an oxygen cylinder for just one patient per year exceeds the initial capital cost of a concentrator. The cost of using an OC for one patient for a year is 27% of the cost of using one cylinder, and if there are 4 patients per year, the cost is 7% of the cost of using the cylinder
Howie et al., 2009 Gambia [50]	To compare oxygen supply options for healthcare facilities and develop a decision algorithm for the selection of oxygen supply systems in Africa and the rest of the developing world	OCs have significant advantages compared to cylinders where the power supply is reliable; In other environments, cylinders are preferable if transport is possible. Cylinder costs are heavily influenced by leakage, which is a common occurrence, whereas concentrator costs are influenced far more by electricity costs than capital costs. Only two of twelve facilities in The Gambia were found to be suitable for concentrators; In the remaining 10 systems, cylinders were the better option
Dobson., 2001 LRSs [51]	To compare the logistics and costs of two systems of oxygen supply for clinical use: oxygen cylinders and OC	The study found that while oxygen cylinders are widely used, logistical and cost factors often favour the use of OCs, particularly in many developing countries where cylinder supplies are inadequate or unavailable. OCs, electrically powered devices that produce high concentrations of oxygen from indoor air, are used successfully in various countries
Periled et al., 2004 Senegal [52]	To evaluate the use of OCs in a rural Senegalese hospital for children who met one or more of the WHO clinical oxygens	The main finding of the study was that the use of OCs was both clinically and financially beneficial and highlighted the need for specific training of the medical team and a strict maintenance program when introducing new technologies

The critical importance of context when selecting oxygen delivery systems for healthcare facilities in LRSs is power availability and logistics costs. While OCs offer significant cost and operational advantages in environments with stable power supply, their dependence on reliable power supply limits their applicability in many environments. Conversely, oxygen cylinders, although often more expensive and prone to leaks, prove to be a more viable option in areas with unreliable power supplies but accessible transportation routes. This nuanced understanding led to the development of a decision algorithm that not only helps make informed decisions but also enables significant cost savings by adapting solutions to specific local conditions. If widely adopted, this approach could increase the efficiency and effectiveness of oxygen delivery in various healthcare contexts in developing countries and ultimately improve patient outcomes [45]. A study conducted in Kenya assessed and analysed the availability of therapeutic oxygen, of which 11 facilities had no oxygen equipment, and nine facilities had at least one concentrator or cylinder. The study also assessed the reliability of the power grid, which has an average performance of seven interruptions per week (range: 2‐147). The average duration of the power outage was 17 min and the longest was more than 6 days [19].

Through a non-experimental before and after study and the availability of oxygen equipment (e.g., respiration masks), two studies carried out in Ethiopia prioritize decentralizing oxygen availability and use at the primary care level for children under five years old with severe pneumonia in 12 health centres in Ethiopia. Concentrators and pulse oximeters) in the facilities; prior to the interventions, seven (58%) of the facilities lacked any; however, as a result of the interventions, every facility had oxygen equipment [46].

Capacity building and technical support measures implemented in 32 public hospitals in Ethiopia resulted in a statistically significant increase in paediatric inpatient wards from 62 to 100% in general and referral hospitals. Out of these 32 establishments, 15 (46.9%) functioned as general hospitals, 10 (31.2%) as referral hospitals, and 7 (21.9%) as primary hospitals. Out of 11 hospitals, only 2 (18%) had a functioning oxygen supply in their paediatric wards. Two concentrators in Uganda produced an O2 stream, while the remaining two did not function at all [47].

The primary power supplies used by these healthcare facilities were also unstable, resulting in frequent power outages that ranged from two to 147 per week and variable amounts of downtime, including one that lasted more than six days. An oxygen supply powered by solar energy turned out to be more affordable than both mains-powered concentrators and no oxygen supply at all. Costs might even be reduced in comparison to concentrators using a fuel generator.

The study [48] evaluated the costs and health effects of solar-powered oxygen supply over a ten-year period with three alternatives, including a zero case without oxygen supply, grid-powered OCs, and fuel generator-powered concentrators. When compared to zero case and grid-powered concentrators, the utilization of a solar-powered oxygen supply proved to be more economical. It also saved money as opposed to employing fuel generator concentrators.

A notable challenge was the lack of trained biomedical personnel for basic OC maintenance, resulting in nurses assuming maintenance responsibilities.

OCs and conventional, manually refilled oxygen cylinders were measured for cost effectiveness in a study by [17, 49]. With the system, oxygen supply costs were reduced by at least 51% as compared to traditional cylinders. The frequent occurrence of cylinder leaks may have contributed to even larger savings. The system was reportedly more dependable, user-friendly, and cost-effective, according to users. Nonetheless, the importance of ongoing staff training and technical support was emphasized.

Cost analysis revealed that providing cylinder oxygen to a single patient over a year exceeded the initial capital investment for a concentrator. In particular, the use of an OC for one patient for one year was only 27% of the costs associated with the use of cylinders. When expanded to four patients per year, the cost of the concentrator fell even further to just 7% of that associated with cylinders, while the cost of maintaining oxygen levels is still to be considered [17].

The advantages of OCs over cylinders were particularly evident in environments with stable power sources. In contrast, in other scenarios where there were concerns about power reliability, cylinders remained the preferred choice as long as transportation was logistically feasible. Cylinder costs were significantly influenced by the occurrence of leaks, which was a common problem, while concentrator costs depended primarily on electricity costs rather than initial capital investments [50].

Cylinders are widely used, logistical and cost factors often favour the use of OCs, especially in LRs where cylinder supplies are insufficient or unavailable. OCs, which are electrically powered devices that generate high concentrations of oxygen from room air, have been successfully used in various countries [51].

The use of an OC has great advantages, especially in LRS, as it helps save money both clinically and financially. Although it offers advantages, the disadvantage is that it is dependent on the power grid and will not work if it is not available due to a continuous power supply problem or an interruption [52].

## Discussions

This systematic literature review allowed the collection and evaluation of the current body of literature related to oxygen concentrators and oxygen systems in SSA. It is noteworthy that the studies currently existing in literature mainly focus on the OC design and availability, as well as their cost effectiveness compared to oxygen cylinders.

Another critical point that was highlighted by this review is that there is a serious maintenance challenge in SSA, due to the different environmental and infrastructural conditions affecting the use and repair of OCs, and MDs in general. It could be noted how most OCs failures could not be resolved at facility level and within the capacity of the locally available biomedical engineers and technicians due to maintenance complexity and the lack of spare parts. The most complex failures of OCs resulted to be compressor wear, molecular sieve depletion, and power control board failure. This underscores the importance of both a strong and well-organised healthcare technology management plan, inclusive of both preventative and corrective maintenance, and of capacity building of biomedical engineers and technicians. In fact, whilst OCs are supposed to have a life span of 7 years or more with proper maintenance, challenging conditions can lead to a greatly decreased life span (i.e., less than 3 years) without intervention [53].

A study conducted by lam et al. best conveyed the effectiveness that oxygen intervention devices had on under-5 s child mortality deaths [54]. By introducing new devices, improving training, supervision and implementing quality improvement approaches, under-5 pneumonia mortality rates reduced from 4.3% to 2.6%. This statistic is reflective of the importance of oxygen delivery devices within LRS. Designing sustainable and resilient devices, as well as investing in capacity building, is, therefore, prevalent for long-term reduction in mortality rates.

Environmental conditions in SSA countries often differ from those to which MD design specifications are tailored and impact the durability of OCs. This requires better adapting OCs and other MDs to the local context and developing solutions that are more resilient to complex conditions [28, 29]. For example, molecular sieves and power control boards are very sensitive to humidity and power fluctuations, which are quite frequent in SSA. Similar considerations are presented in the WHO “Compendium of innovative health technologies for low-resource settings” series.

In this regard, in this review, it was also found that solar-powered oxygen systems have emerged as a cost-effective and sustainable solution, particularly in rural areas with unreliable power grids. These systems provide a reliable source of oxygen and can significantly reduce operating costs. However, the high initial capital investment remains a challenge that needs to be addressed for widespread adoption. It is noteworthy that the potential global impact from these systems extends beyond their immediate benefits, offering a sustainable solution that aligns with the United Nations’ Sustainable development goal 7 (SDG7), which promotes affordable and clean energy, by reducing the large carbon footprint that is often associated with MDs. Therefore, designing innovative technologies in a context-driven way can contribute to overall sustainability.

Moreover, these technologies showed great effectiveness as seen in a study in Papua New Guinea [55]. Here the implementation of such devices from having no OCs to gaining solar powered ones resulted in a 20% reduction in overall mortality rates. This emphasises how important and effective oxygen therapy can be in LRS. By implementing this model in more LRS it could provide a more consistent sustainable source of oxygen, increasing trust in and reliability of healthcare providers, especially in more rural locations.

Nonetheless, although this technology shows great promise, it is important to note that financial barriers may still hinder access to these technologies. In fact, they require high capital investment from governments and with more complex components their maintenance demands may also be greater. Research into policy interventions addressing these issues will have to be considered first before implementing these technologies in LRS.

### Limitations

This study has potential limitations as with any study. This study only assessed 20 papers due to the limited number of evidence available in this research area. The papers selected were from a multitude of diverse sources, however, collectively they attempted to full encapsulate the research area. It is noteworthy that given the span of this review (more than 20 years), it may be that it collects evidence from different types of OCs, developed with different technological advancement. This can have a direct impact on the way they react to the local challenges presented so far. Publication bias is also important to consider with this systematic review: due to the tendency of publishing studies with positive or significant results, it may be that some evidenced has been missed out, giving a skewed and distorted view of the research area. Finally, even if our search strategy attempted to be as inclusive and complete as possible in order to discover relevant articles in the topic area, it might have missed some articles, including especially those that are hosted on different databases. However, our selection of the most widely used databases for this type of research should reassure the reader in this aspect.

### Recommendations

Based on the results of this systematic literature review, we recommend a comprehensive approach to address the challenges related to oxygen supply and conservation in SSA. These recommendations aim to improve the reliability and availability of oxygen therapy in healthcare facilities across the region:

1. Implement solid maintenance plans, inclusive of:
  Clear maintenance intervals: Create standardized schedules for preventive maintenance (e.g., timely compressor performance check according to the service manual).
  Training Protocols: Develop and disseminate training modules for biomedical engineers and technicians, focused on troubleshooting common failures (e.g., compressor wear, molecular sieve exhaustion) and performing complex repairs.
   Spare part management: Create regional spare parts storage and distribution centres to ensure timely access to critical components such as molecular sieves, compressors, and power control boards.
  Documentation and monitoring: Implement a digital tracking system to log maintenance activities, equipment performance, and maintenance history, to enable data-driven decision making.

2. Adapt/Re-design technology for local contexts. To address environmental challenges such as humidity, temperature fluctuations, and power instability, the following steps are recommended:
  Local collaborations: Collaborate with local engineers and manufacturers to develop and test OCs tailored to SSA’s environmental challenges.
  Develop standardized test protocols: Create context-specific test protocols to assess device durability under simulated local conditions (e.g. high humidity, power surges).
  Design Improvements: Incorporate features such as moisture-resistant molecular sieves and surge-protected power control boards to improve device resilience.

3. Promoting the dissemination and uptake of solar-powered oxygen systems. Solar-powered oxygen systems provide a sustainable solution for rural and off-grid areas. To promote their adoption:
  Explore financing mechanisms: Develop innovative financing models such as public–private partnerships, international grants or community-based financing to reduce the high initial capital investments.
  Conduct feasibility studies: Evaluate the feasibility of solar-powered systems in various SSA regions, considering factors such as sunlight availability, maintenance requirements, and cost effectiveness.
  Providing Training: Training biomedical engineers and technicians in the installation, operation and maintenance of solar powered systems to ensure their long-term sustainability.

4. Strengthening the locally available expertise: Invest into capacity building for the development of locally available teams of experts able to effectively use and maintain oxygen systems. Recommendations include:
  Training Programs: Develop and implement training programs for biomedical engineers, technicians, and healthcare workers with a focus on equipment operations, maintenance, and troubleshooting.
  Certification Systems: Establish certification programs to ensure trained professionals meet standardized levels of competency.
  Knowledge sharing: Create knowledge sharing platforms, e.g., regional workshops, maintenance book and online forums and technical manuals to disseminate best practices and innovations.

5. Improve supply chain management. An uninterrupted supply of oxygen devices and spare parts is essential for long-term functionality. Key measures include:
  Local manufacturing: Promote local production of critical components such as molecular sieves and compressors to reduce dependence on international supply chains.
  Regional distribution networks: Establish regional hubs for storage and distribution of spare parts to ensure timely access and reduce downtime.
  Inventory management systems: Encourage to use medical equipment management system (MEMS) to track inventory levels, predict demand, and automate reordering processes.

6. Develop supportive policies and regulatory frameworks. Governments and stakeholders should create an enabling environment for the sustainable use of oxygen systems by:
  Regulatory Standards: Establishing and enforcing standards for the quality, safety and performance of OCs and related equipment in LRSs.
  Fund Allocation: Allocate dedicated budgets for oxygen therapy infrastructure, including equipment procurement, maintenance, and training programs.

7. Ensuring Equitable Access to Oxygen Therapy To address disparities in access, particularly for marginalized and underserved communities, the following strategies are recommended:
  Community involvement: Include local communities in the planning and implementation of oxygen therapy programs to ensure they are specific meet needs and cultural contexts.
  Outreach programs: Deploy mobile oxygen units or community-based oxygen stations to reach remote and underserved areas.
  Targeted Interventions: Prioritize vulnerable populations such as children, pregnant women, and the elderly in oxygen therapy initiatives.

By implementing these recommendations, governments, health authorities and stakeholders can help improve the accessibility and effectiveness of medical oxygen systems in SSA. This, in turn, will have a direct and positive impact on healthcare outcomes, ultimately saving lives and strengthening healthcare systems in the region.

## Conclusions

In conclusion, this systematic review highlighted the significant challenges in the design and maintenance of medical OCs in SSA, emphasising the need for more comprehensive research into addressing healthcare limitations.

The study showed that whilst OCs are crucial in reducing mortality rates and extremely effective in combatting respiratory diseases, their effectiveness is constantly hampered by maintenance issues, environmental factors and supply chain problems. It found that the most complex of failures came from compressor wear, molecular sieve depletion, and power control board failure. Underlining the need for robust maintenance plans and having capacity building in place.

Solar power OCs showed a large amount of potential within concentrator developments, as access to renewable power within the device helped to eliminate power supply issues that regularly hinder concentrators effectiveness in LRS, especially within rural areas. These systems not only address power supply challenges but also align with SDGs by reducing the carbon footprint from medical technologies.

This review recommends multiple approaches to address these challenges including; promoting local manufacturing of critical components; adapting the technologies in a frugal way to better fit the specific local environments; promoting solar powered OCs especially to rural areas; and finally implementing better quality assurance processes during acquisition. By focusing on these strategies, healthcare providers can significantly improve their oxygen reliability and accessibility in LRS.

Future research should prioritise conductions studies investigating the long-term cost effectiveness and efficiency of different types of oxygen generators in SSA. More research is needed surrounding the technology in OCs for LRS, this should be carried out in a context-driven way, prioritising developing more durable materials and components. Investigations into MDs for military use could be a good starting point, as they often encounter similar environmental challenges in deployment but without the financial constraints of LRS.

Finally, creating policies and frameworks that incentivize local manufacturing and technology adaptation for LRS could have a greater long-term sustainability for MDs, removing the reliance from imports.

## Supplementary Information

Supplementary Material 1.

Supplementary Material 2.

## Data Availability

All data generated or analysed during this study are included in this published article and its supplementary information files.

## Abbreviations

SSA: Sub Saharan Africa
LRS: Low resource setting
PCB: Printed circuit board
MMAT: Mixed Methods Appraisal Tool
LMIC: Low- and middle-income countries
WHO: World Health organization
MD: Medical devices
OC: Oxygen concentrators
